# *Aphanomyces macrosporus* sp. nov. Causing Root Rot in Barley and Some Other Plants

**DOI:** 10.3390/jof9121144

**Published:** 2023-11-27

**Authors:** Mariann Wikström, Lars Persson, Jamshid Fatehi

**Affiliations:** 1 Agro Plantarum AB, Kärrarpsvägen 410, S-265 90 Åstorp, Sweden; 2 Brandsberga Gård AB/Agri Science Sweden AB, Brandsberga Gård 210, S-264 53 Ljungbyhed, Sweden; 3 Lantmännen BioAgri, Fågelbacksvägen 3, S-756 51 Uppsala, Sweden; jamshid.fatehi@lantmannen.com

**Keywords:** *Aphanomyces* sp., Sweden, Denmark, root disease, *Hordeum vulgare*, *Spinacia oleracea*, *Beta vulgaris*

## Abstract

In recent years, a new root rot disease in barley, which is caused by an *Aphanomyces* species, was found in field surveys in Southern Sweden and Denmark. Its symptoms occurred at the early tillering stage, around the BBCH 21 growth stage, and included the yellowing of leaves, brown coleoptiles, and the discolouration of roots. Prolonged soil wetness after rainfall favoured disease development, which sometimes advanced the yellowing patches to entire fields, resulting in lower yields. Oospores were found in the fine roots of diseased plants, and *Aphanomyces* isolates were obtained from these roots, as well as from the roots of barley plants grown in the greenhouse in soil samples from infected fields. Based on morphological analysis, we found that the new isolates were similar to those already obtained from barley and spinach roots in the 1990s in the same growing area. The morphological and molecular analyses performed in this study clearly separated and distinguished these barley isolates from other known *Aphanomyces*, and hereby *Aphanomyces macrosporus* sp. nov. is proposed as a new plant pathogenic species. It has larger oogonia and oospores than *A. euteiches*, *A. cochlioides*, and *A. cladogamus*, with one up to eight diclinous antheridia per oogonium. The phylogenetic analysis of the ITS rDNA region sequences grouped these new *Aphanomyces* isolates in a monophyletic clade, which was clearly distinguished from other plant pathogenic *Aphanomyces* species. The further pathogenicity of *A. macrosporus* on other plants is currently under investigation, but it is clear that it can at least infect barley, spinach, and sugar beet, indicating a wide host range for this species. The widespread presence and presumably broad host range of this new pathogenic *Aphanomyces* species must be considered in crop rotations.

## 1. Introduction

The genus *Aphanomyces* includes several species that are highly parasitic on plants and animals and also a few saprotrophic species growing on plant and animal debris [1,2]. Among the most important plant pathogenic species are *Aphanomyces euteiches* Drechsler, causing root rot in peas; *A. cochlioides* Drechsler; and *A. cladogamus* Drechsler, causing damping off in sugar beet and spinach [3,4,5,6,7,8,9,10]. These diseases are favoured by wet conditions and high rainfall, especially in the beginning of the growing season after sowing. Symptoms are often most prevalent in the lower parts of fields with high soil water content. Oospores are produced in infected roots and can survive for several years in infested soil, maintaining high root rot potential. For crops such as pea, sugar beet, and spinach, it is possible to assess root rot potential in advance prior to sowing to avoid severely infested fields and yield losses. Common symptoms in fields infected with *Aphanomyces* include the yellowing of plants and eventual wilting due to the restricted transport of water and nutrients. 

In a previous study by one of the authors of this article, M. Wikström, formerly Larsson [11], several isolates of an unidentified species of *Aphanomyces* were isolated from spinach and barley roots, and their pathogenicity on these host plants was approved using Koch´s postulates. Analyses using isozymes indicated that they were different from *A. euteiches*, *A. cochlioides*, and *A. cladogamus*, and therefore it was suggested that they could belong to a new *Aphanomyces* species. However, no name was proposed at that time. Later on, in 2011, a barley field with stunted plants was found in the province of Västergötland, where the field was overgrown by weeds, and the yield was very low. A laboratory examination of the infected plant roots resulted in an isolate of the *Aphanomyces* species, which was morphologically similar to the unknown *Aphanomyces* isolates detected in the 1990s. 

In 2021, heavy rains in the spring after sowing induced wilting and yellowing patches in many barley fields in the south of Sweden, resembling the symptoms seen in pea, sugar beet, and spinach fields infected with *Aphanomyces*. Sometimes, plants died; the yield in infected barley fields was at least 20% below the expected yield, and the crop was premature. Field surveys revealed that oospores were present in the fine roots of the diseased plants, and isolation from these roots resulted in *Aphanomyces* cultures which were morphologically similar to the unidentified *Aphanomyces* species discovered in the 1990s and in 2011.

The discovery of the natural infection of *Aphanomyces* in barley fields and consequent yield loss as a result of the disease is, to our knowledge, new. Moreover, the pathogenic isolates from barley are morphologically and molecularly distinguishable from the other common plant pathogenic *Aphanomyces* species, suggesting that they belong to a new and undescribed species. The aim of this study is to identify, characterise, and describe the *Aphanomyces* species causing infection and root disease in barley. 

## 2. Materials and Methods

### 2.1. Field Surveys and Pathogen Isolation

During the growing season in 2021, there were heavy rainfalls in the western part of Scania and the southern Halland provinces in Sweden. In that period, several barley fields where the plants had turned yellowish and stunted were found. The discoloured roots from yellow plants collected from several fields were examined under the microscope for the detection of the possible presence of oospores. If oospores resembling *Aphanomyces* spp. were detected, small root pieces were plated on *Aphanomyces* selective medium (SMA), as described by Larsson and Olofsson [5]. In addition, as a standard procedure, all root pieces were also plated on potato dextrose agar (PDA; Difco^TM^, BD Diagnostics, Erembodegem, Belgium) amended with the antibiotic streptomycin sulphate (100 mg L^−1^) at room temperature to isolate possible *Rhizoctonia*, *Fusarium*, *Pythium*, or other root pathogens. Soil samples were also taken from those barley fields and from some other fields in Västergötland in the west of Sweden and from Jutland in Denmark. Each sample was divided between two 1 L pots, and 10 barley seeds were sown in each pot. The pots were placed in a greenhouse equipped with high-pressure sodium lamps (Osram, HQI-T 400W, Munich, Germany) under a 16/8 h day/night photoperiod at 24/19 °C. After four weeks, the barley plants and roots were washed and examined. If there were any visible symptoms, the roots were examined under the microscope, and small root pieces were plated on the SMA medium. Growing colonies were checked under the microscope, and those resembling *Aphanomyces* cultures were transferred to corn meal agar (CMA; Difco^TM^, BD Diagnostics, Erembodegem, Belgium). Hyphal tip cultures were established for all the isolates, and they were then stored on CMA at 4 °C.

### 2.2. Pathogenicity

The pathogenicity tests of three isolates, B8, B9, and B11, were performed on barley cv. Planet under greenhouse conditions in order to examine their ability to cause disease symptoms similar to those observed in the field. In addition, other plants, such as spinach cv. Kiowa, sugar beet cv. Cartoon, peas cv. Linnea, and faba beans cv. Alexia, were included in the pathogenicity tests to repeat and to verify our findings from the earlier studies [11,12]. Radish (*Raphanus sativus*), cv. Cherry Belle, was also included in the pathogenicity assay to compare the pathogenic properties of the isolates to those of *A. raphani* J.B. Kendr., capable of infecting *Raphanus* spp. [13]. Briefly, the strains were cultivated on CMA for 10 days; then, the agar plates with the cultures were placed as an inoculum layer in pots filled with vermiculite. Before sowing, the seeds were surface-sterilised in a sodium hypochlorite solution consisting of 1 part common laundry bleach (2.7% sodium hypochlorite) to 2 parts clear water for 2 min and were then thoroughly rinsed in water. Seeds were placed one centimetre above the inoculum layer. The pots were placed in a greenhouse with similar conditions to those described above. Four weeks after sowing, the roots were washed in water and the symptoms were checked. Small root pieces with discoloured roots were placed on selective agar medium (SMA) for re-isolation of *Aphanomyces* sp. The plates were checked daily for outgrowing colonies.

### 2.3. Morphology

Colony morphology on CMA and PDA was continuously evaluated for 14 days of growth at 20 °C. The colony features, such as presence of aerial mycelium, colony edge shape, and presence of floral patterns, were recorded. The dimensions of oospores and oogonia and the hyphal diameters of CMA cultures from 10 different isolates were measured using an Olympus microscope (Evident cooperation, Tokyo, Japan) equipped with an INFINITY1 Digital CMOS Color Microscopy Camera (Teledyne Lumenera, Ottawa, ON, Canada) and INFINITY ANALYZE 7 software. Twenty oospores and oogonia per isolate were measured. The oogonia and oospore measurements were compared with our old data from seven isolates from barley and spinach published previously by Wikström [11]. Sporangial formation was induced in sterile tap water poured onto the 5-day-old growing culture of B8, B9, and B11 isolates on CMA at room temperature. The plates were examined under the microscope after 16–48 h for the presence of zoosporangia and zoospores.

### 2.4. Temperature Response

To determine the minimum, maximum, and optimal growth temperatures, four isolates, B8, B9, B11, and B21, were included in these experiments. Fungal plugs 5 mm in diameter obtained from the margins of 5-day-old cultures were inoculated at the centre of CMA plates, with 2 replicates per isolate. Cultures were incubated at 1.0, 3.0, 3.5, 6.0, 11.0, 13.0, 17.0, 20.5, 21.0, 24.5, 27.0, 30.0, 32.5, and 34.0 °C. Radii of the colonies were measured at four readings per plate after 24 h.

### 2.5. Molecular Characterisation

Mycelial plugs of young cultures of 12 different *Aphanomyces* isolates on PDA were transferred into Petri plates containing 25 mL of 0.1× strength PD broth under stationary conditions for 5–6 days at 22–24 °C. Approximately 200 mg of growing mycelia were separated from the agar plugs with a sterile scalpel and transferred into a 1.5 mL microcentrifuge tube. They were first washed with sterile water and the total genomic DNA was extracted using the GeneJET Plant Genomic DNA Purification Mini Kit (Thermo Fisher Scientific, Baltics UAB, Vilnius, Lithuania) according to the manufacturer’s instructions. The PCR amplification of the region of the nuclear ribosomal DNA that included ITS1, 5.8s, and ITS 2 was carried out with ITS6 and LSU-0344R primers using DreamTaq Hot Start Green DNA Polymerase (Thermo Fisher Scientific, Baltics UAB, Vilnius, Lithuania) according to the conditions described by Levenfors and Fatehi [12]. The PCR products were purified using a GeneJet PCR purification kit (Thermo Fisher Scientific, Baltics UAB, Vilnius, Lithuania), and they were sequenced in both directions at the sequencing facilities of Macrogen Europe (Amsterdam, The Netherlands). 

Sequences of *Aphanomyces* isolates obtained in this study were manually edited and proofread in the DNAStar software package (version 5.07 Lasergene, Madison, WI, USA). They were added into a sequence data set available from our previous *Aphanomyces* study [12] and were aligned using the ClustalW method in the MegAlign program of the same software. Moreover, ITS sequences of several other *Aphanomyces* species available from GenBank were included in this sequence alignment. Sequences were deposited at the National Center for Biotechnology Information (NCBI) GenBank, and the accession numbers are listed in Table 1.

Maximum parsimony (MP) analysis of the ITS data was performed with PAUP* version 4.0 [14] using 10,000 replicates of a heuristic search with the random addition of sequences and subsequent TBR branch swapping with no limits of rearrangement, and alignment gaps were treated as missing data. The trees were rooted with the sequence data of the ITS region from *Saprolegnia parasitica* and *S. diclina* [15,16]. The robustness of the internal branches was assessed via bootstrap (BS) analysis with 1000 replications in a heuristic search with 10 rounds of random sequence addition and subsequent TBR branch swapping.

For the Bayesian analysis of the ITS sequences, first, the alignment data were analysed in Mr.Modeltest 2 [17] to select the appropriate model of DNA substitution with the hierarchical likelihood ratio test (hLRT) implemented in the program. The best-fit selected model (by hLRT) was the general time-reversible substitution model (GTR) [18], which was used in subsequent Bayesian analysis. MCMC was performed with the computer program MrBayes 3.2.7 [19]. Four incrementally heated simultaneous Markov chains were run for 5,000,000 generations, from which every 100th tree was sampled.

The initial 5000 saved trees were discarded to ensure that only trees that were created after reaching a stable log-likelihood value were included. From the remaining 45,000 trees, a 50% majority-rule consensus tree was computed, and the posterior probabilities of the groups were estimated.

**Table 1:** The GenBank accession numbers, hosts, and geographical origins of *Aphanomyces* species used for phylogenetic analysis.

Scientific Name	GenBank Accession Number	Host	Geographical Origin	Reference
*A. macrosporus* B8	OQ972001	*Hordeum vulgare*	Sweden	This study
*A. macrosporus* B9	OQ972002	*Hordeum vulgare*	Sweden	This study
*A. macrosporus* B10	OQ972003	*Hordeum vulgare*	Sweden	This study
*A. macrosporus* B11	OQ972004	*Hordeum vulgare*	Denmark	This study
*A. macrosporus* B12	OQ972005	*Hordeum vulgare*	Sweden	This study
*A. macrosporus* B13	OQ972006	*Hordeum vulgare*	Sweden	This study
*A. macrosporus* B14	OQ972007	*Hordeum vulgare*	Sweden	This study
*A. macrosporus* B15	OQ972008	*Hordeum vulgare*	Sweden	This study
*A. macrosporus* B17	OQ972009	*Hordeum vulgare*	Sweden	This study
*A. macrosporus* B18	OQ980615	*Hordeum vulgare*	Sweden	This study
*A. macrosporus* B19	OQ972010	*Hordeum vulgare*	Sweden	This study
*A. macrosporus* B20	OQ980616	*Hordeum vulgare*	Sweden	This study
*A. macrosporus*	AY353921	*Phaseolus vulgaris*	Sweden	[12]
*A. cladogamus*	AY353913	*Medicago sativa*	Sweden	[12]
*A. cladogamus*	AY353918	*Spinacia oleracea*	Sweden	[12]
*A. trifolii*	GQ267548	*Trifolium subterraneum*	Australia	[20]
*A. trifolii*	GQ267549	*Trifolium subterraneum*	Australia	[20]
*A. cochlioides*	FM999224	*Beta vulgaris*	Sweden	[15]
*A. cochlioides*	AY353911	*Beta vulgaris*	Sweden	[12]
*A. raphani*	MK513783	Fresh water	Brazil	[21]
*A. euteiches*	AY353901	*Pisum sativum*	Sweden	[12]
*A. euteiches*	AY353902	*Pisum sativum*	USA	[12]
*A. euteiches*	AY353906	*Vicia arvense*	Sweden	[12]
*A. euteiches*	AY353907	*Medicago sativa*	USA	[12]
*A. euteiches*	AY353908	*Medicago sativa*	USA	[12]
*A. euteiches*	AY353909	*Phaseolus vulgaris*	USA	[12]
*A. euteiches* f. sp. *phaseoli*	AY353910	*Phaseolus vulgaris*	USA	[12]
*A. iridis*	HQ643121	*Iris hollandica*	Japan	[22]
*A. invadans*	FM999229	*Brevoortia tyrannus*	USA	[15]
*A. invadans*	FM999231	*Brevoortia tyrannus*	USA	[15]
*Saprolegnia diclina*	KM061644	Water	Falkland Islands	NCBI Genbank
*Saprolegnia parasitica*	AY455776	*Salmo trutta*	Outgroup	NCBI Genbank

## 3. Results

### 3.1. Pathogen Isolation, Disease Symptoms, and Pathogenicity

**Figure 1:**
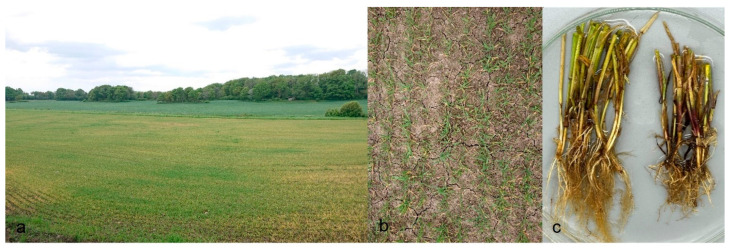
(**a**,**b**) Barley field with infection of *Aphanomyces macrosporus* causing yellow patches. (**c**) Moderately and severely infected field plants with brown coleoptiles and discoloured roots.

**Figure 2:**
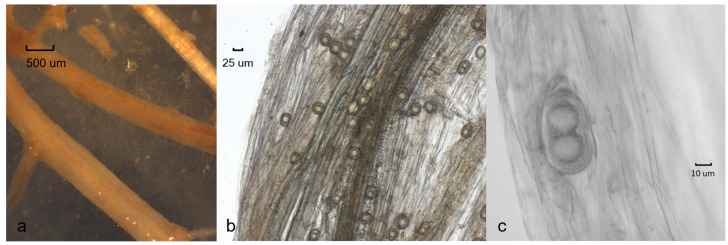
Symptoms of barley roots with infection of *Aphanomyces macrosporus*: (**a**) fine roots; (**b**) oospores in root tissue; (**c**) double oospores.

Field surveys revealed that the *Aphanomyces* pathogenic on barley is widespread in the agricultural areas of Southern Sweden. Isolates were obtained from several agricultural fields in the southwest part in Scania, Halland, and Västergötland, and also in Jutland in Denmark. In total, around fifty isolates were retrieved from barley fields. In early symptoms around BBCH 21, the leaves of diseased barley plants had turned yellow, the coleoptiles were brown, and the roots were slightly discoloured (Figure 1a–c). Infected root tips with minor lesions were occasionally found (Figure 2a). Typical *Aphanomyces* oospores were found in infected root tissues examined under the microscope (Figure 2b,c). 

**Figure 3:**
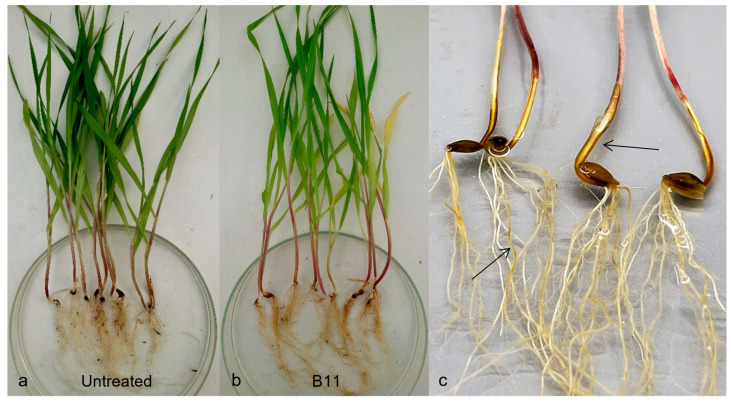
Symptoms on barley roots after inoculation with *Aphanomyces macrosporus*: (**a**) uninoculated healthy plants; (**b**,**c**) inoculated plants with brown coleoptiles and discoloured roots (indicated by arrows).

**Figure 4:**
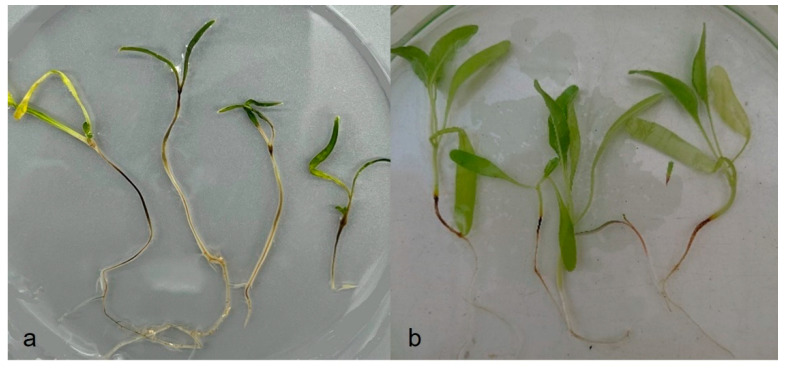
Infected plants with brownish hypocotyls and roots after inoculation with *Aphanomyces macrosporus:* (**a**) spinach; (**b**) sugar beet.

**Figure 5:**
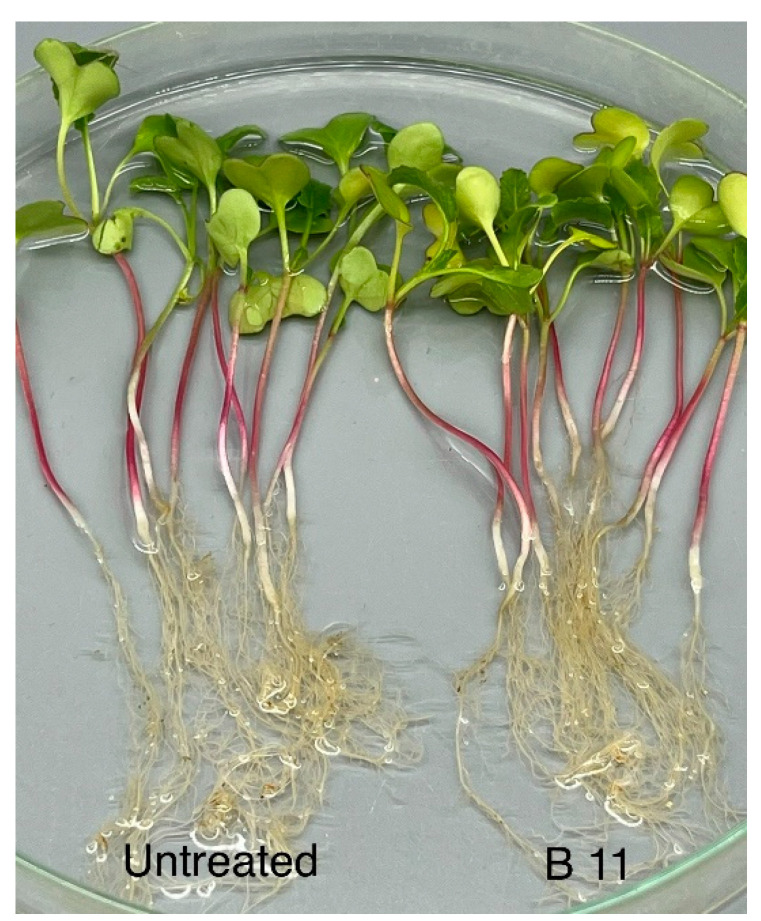
*Aphanomyces macrosporus* isolate B11 inoculated on radish plants to the right in comparison with untreated control plants to the left.

In artificial pathogenicity experiments using the inoculum layer method, disease symptoms like those observed in the field were developed in barley, spinach, and sugar beet (Figure 3a–c and Figure 4a,b). No symptoms at all were found in peas or faba beans (data to be published). In the case of spinach and sugar beet, the symptoms resembled those caused by *A. cladogamus* and *A. cochlioides* infection, i.e., seedling damping off, as well as plants with brownish hypocotyls and roots [11]. Isolations of root segments on selective agar media and observations of growing colonies of *Aphanomyces* verified the pathogenicity and fulfilled Koch’s postulates. A similar pathogenicity assay with radish plants resulted in no symptoms at all on the plants, indicating that the barley *Aphanomyces* isolates do not belong to *A. raphani* (Figure 5).

### 3.2. Temperature Limits and Optima 

**Figure 6:**
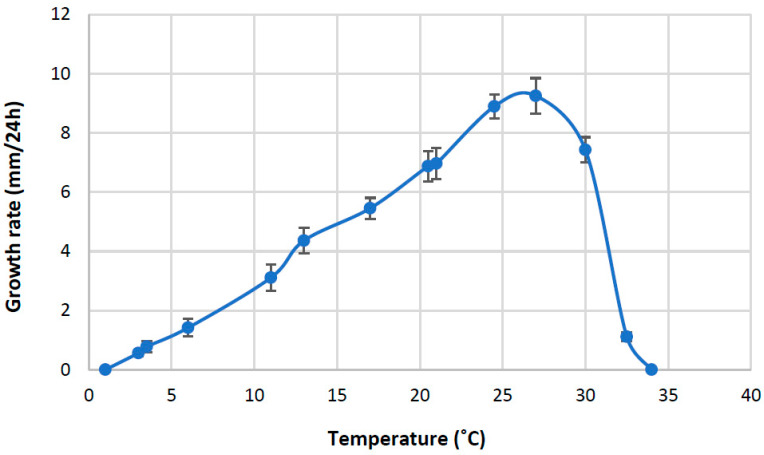
Growth–temperature relationships of *Aphanomyces macrosporus*. Each point represents the average growth rate per 24 h of four isolates with two replicates. Bars represent standard deviations of the means.

No isolates grew at 1 °C or 34 °C. From 3 °C to 25 °C, the increase in growth rate was almost linear (Figure 6), and the optimal growth temperature was 27 °C, followed by a rapid decline at higher temperatures. 

### 3.3. Phylogenetic Analysis

ITS nuclear rDNA sequences of 12 *Aphanomyces* isolates originating from the roots of barley were generated in this study. The sequences of these isolates were identical but different from other known *Aphanomyces* species available in GenBank. In the NCBI BLAST search, the only exception was a single isolate of *Aphanomyces* sp. (accession no. AY353921) isolated from common bean (*Phaseolus vulgaris*) in Sweden [12], which contained an identical ITS to the barley isolates. The ITSs of the novel *Aphanomyces* isolates were aligned with the ITS sequences of the representative plant pathogenic *Aphanomyces* species as well as *A. invadans*, representing an animal-parasitic species. The alignment data contained 642 total characters, of which 415 were constant and 193 were parsimony-informative.

**Figure 7:**
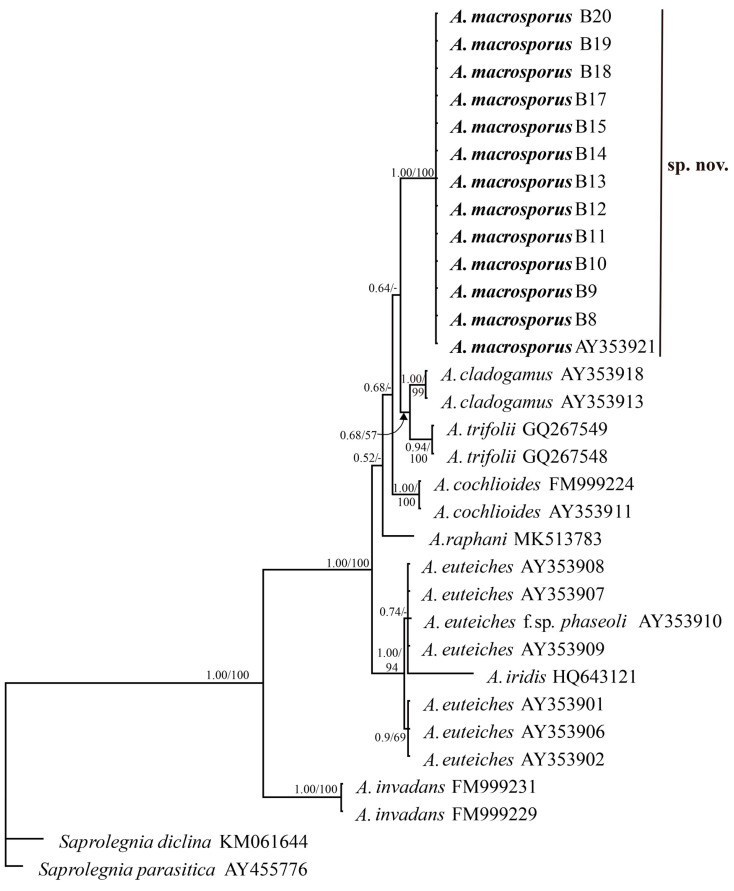
The Bayesian 50% majority-rule consensus tree obtained from the analysis of the ITS sequence data including ITS1, ITS2, and 5.8s rRNA gene. Numbers on the branches are Bayesian posterior probability/parsimony bootstrap values (>50%).

MP analysis of the ITS data resulted in 154 most-parsimonious trees with a score of 344. The barley isolates and the accession AY353921 formed a distinct clade separated from all other *Aphanomyces* species and supported with a bootstrap value of 100%. Similarly, in the Bayesian analysis, this clade was also strongly supported with the highest posterior probability of 1. The majority-rule consensus tree from the Bayesian analysis is shown in Figure 7. While all the *Aphanomyces* species clades were strongly supported in both Bayesian and MP analyses, the posterior probabilities of the positions of the species relative to each other were slightly higher than the MP bootstrapped values.

On the basis of the molecular and morphological characteristics, the *Aphanomyces* sp. causing root disease on barley, spinach, and sugar beet, is considered to be a new species, and therefore we propose the name *Aphanomyces macrosporus* sp. nov.

### 3.4. Taxonomy 

**Figure 8:**
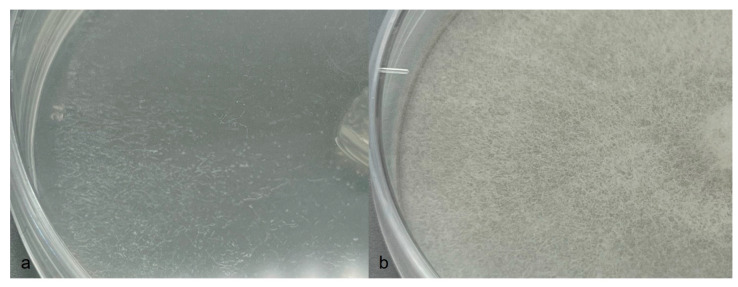
*Aphanomyces macrosporus* on (**a**) CMA to the left and (**b**) PDA to the right.

**Figure 9:**
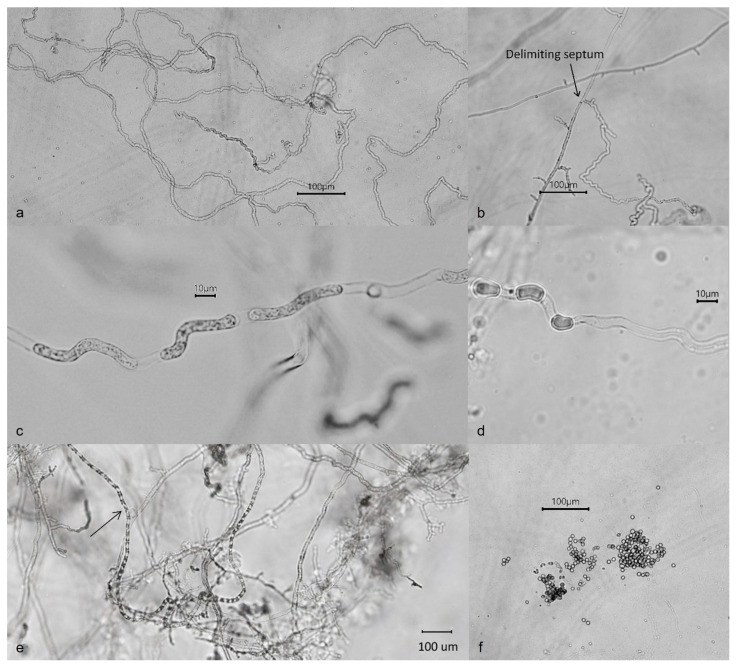
Sporangia of *Aphanomyces macrosporus*: (**a**) long sinuous sporangia; (**b**) delimiting septum between the hyphae and sporangium; (**c**–**e**) sporangia with elongated and moving zoospores immediately preceding discharge; (**f**) primary zoospores encysting at sporangium orifice on emergence.

**Figure 10:**
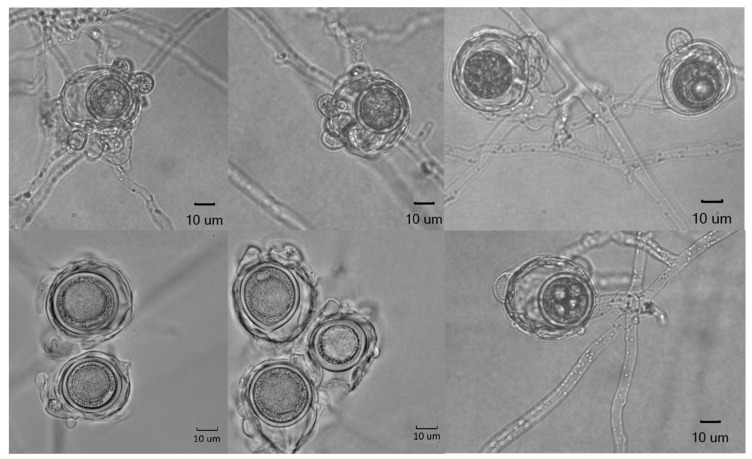
Oogonia, antheridia, and oospores of *Aphanomyces macrosporus*.

*Aphanomyces macrosporus* M. Wikström, L. Persson, & J. Fatehi, sp. nov. (Figure 6, Figure 7, Figure 8, Figure 9 and Figure 10), MycoBank number: MB849876. **Holotype**: Sweden, province of Scania, Kärrarp, isolated from naturally infected roots of *Hordeum vulgare* in field, 16 April 2021. Collector: M. Wikström. K-M1436392 (dried culture). **Ex-type**: Isolate B9 (Agro Plantarum Culture Collection). Gene Sequence ex-type: OQ972002 (ITS). Etymology: “macrosporus” refers to the large oogonia and oospores. 

Colonies on CMA uniform, lacking aerial mycelium, smooth at edge with no floral pattern. On PDA similar to CMA but with denser appearance and presence of aerial mycelia (Figure 8a,b). Growth min. at 1–3 °C, opt. 25–27 °C, max. 32–34 °C. Hyphae hyaline, slender, sparingly or moderately branched and coenocytic, 3.5 to 12 µm (av. 6.4 µm) in diameter. Zoosporangia readily formed in water from CMA culture plugs and distinguished from hyphae with delimiting septa (Figure 9b), sinuous, up to 2 mm or longer in length, with many lateral branches, and not tapering towards apex (Figure 9a,b). Zoospores elongated in the discharge tube, forming spherical cysts (Figure 9c–f) 7.5–9 µm in diameter when discharged; highly abundant, from a few to over 300 clumping together after encystment at sporangium orifice (Figure 9f). Biflagellate and motile zoopores rarely formed. Oogonia (Figure 10 and Table 2) terminal on short or long stalks, subspherical or rarely spherical, smooth, highly variable in size among isolates, 23–60 µm (av. 35 µm) in diameter. Oospores single or rarely double, spherical, often eccentric, with granular contents and a large central oil globule (Figure 10), 15–40 µm (av. 26 µm) average. Antheridia diclinous, from 1 up to 8 per oogonium (Figure 10).

## 4. Discussion

For a long time, the development of yellowing patches in barley fields has been observed in Sweden, particularly after heavy rainfalls during the early cropping season (Figure 1a–c). Indeed, the first report on this observation was published almost 30 years ago by M. Wikström [11]. In that study, several *Aphanomyces* isolates were obtained from barley and one from spinach, which differed from other known plant pathogenic *Aphanomyces* species in their distinct morphologies, pathogenicity, and unique isozymes profile. It was suggested that these isolates could belong to a new *Aphanomyces* species; however, no name was proposed. Our new surveys during 2021–2022 from the same farms sampled 30 years ago, as well as several other new locations in Southern Sweden and Denmark, confirmed the presence of this new *Aphanomyces* species associated with barley root rot. Our findings so far have shown the relatively broad distribution of this pathogenic *Aphanomyces* in Southern Sweden, and currently more surveys are underway to determine its geographic distribution in Sweden. 

The isolation of *Aphanomyces* from infected plant materials can sometimes be difficult, and it often requires the use of selective media and working experience with this pathogen. Therefore, it is not unlikely that *A. macrosporus* has been overlooked in many investigations on barley root rot diseases in Sweden and perhaps in other countries, at least in the Nordic region. When plants from yellow patches were studied, the roots were washed and observed under the microscope and also added to agar media, where *Rhizoctonia*, *Pythium*, and *Fusarium* spp. can also grow. The seeds in commercial fields are most commonly treated with fludioxonil, which is active against *Fusarium* and *Rhizoctonia* [23], and only low levels of these pathogens have been found at this growth stage. *Pythium* may sometimes be present, together with *A. macrosporus*, but in our location normally causes damping off instead of later symptoms like those described in other areas [24]. In pathogenicity tests with *A. macrosporus*, damping off seldom occurred. Since the symptoms of yellow patches appeared in wet soil conditions and *A. macrosporus* was present and repeatedly isolated from the diseased plants, while there were no other pathogens causing similar symptoms, we conclude that *A. macrosporus* is the major cause of barley root rot in those fields.

Among the known plant pathogenic *Aphanomyces* species, *A raphani* may be the only species morphologically close to *A. macrosporus*, by having large oogonia and oospores. However, our isolates have wider ranges of oogonia and oospore dimensions, 23–60 µm and 15–40 µm, respectively, compared to those ascribed to *A. raphani*, 27–52 µm and 19–39 µm, respectively [1,25]. The other two most phylogenetically related species, *A. cladogamus* and *A. trifolii* O’Rourke et al. (Figure 1), can reliably be distinguished from *A. macrosporus*. Both these other species have smaller oogonia and oospores: *A. cladogamus*, 19–33 µm and 15–26 µm, respectively [25]; *A. trifolii*: 24–31 µm and 17–23 µm, respectively [20]. Moreover, the number of antheridia per oogonium in *A. raphani* has been reported to range from one to a maximum of three [13,25], while in *A. macrosporus* we have observed from one up to eight attached antheridia per oogonium. Furthermore, the pathogenicity of our isolates did not in any way match that of *A. raphani*. According to Scott [1], *A. raphani* is parasitic on radish, *R. sativus*, where it causes serious disease. Hall [26] reports that host species of *A. raphani* occur only in the family *Cruciferae*. Our isolates were found on barley and spinach roots, and they did not cause any symptoms at all on radish. Unfortunately, isolates of *A. raphani* was not available in culture collections for comparison, but the only single ITS accession number available for this species, recently published by Pires-Zottarelli et al. [21], was included in the phylogenetic analysis in this study, and it was clearly distinct from *A. macrosporus*. 

The identification of *Aphanomyces* isolates has been greatly facilitated using ITS sequencing data, which has overcome the difficulties and challenges of using morphologies, which are often limited by insufficient characters and large overlapping variations of the asexual and sexual structures. In this context, several studies have shown that ITS-based phylogenetic analysis can be reliably used for the identification and differentiation of species, elucidation of genetic relationships, and discovery of new members of the *Aphanomyces* genus [12,15,20,21]. The ITS sequence data of the single isolate of *Aphanomyces* (accession AY353921), originated from the roots of *P. vulgaris*, revealed that it was a distinct species which did not cluster with other plant pathogenic *Aphanomyces* species in the phylogenetic tree [12,15]. The ITSs of barley isolates were all identical to AY353921, which formed a well-supported and distinct cluster in this study. 

So far, our studies have shown that *A. macrosporus* can cause root rot disease symptoms in barley, spinach, and sugar beet, and it is not pathogenic on peas, faba beans, and radish [11] (unpublished data). Similarly, the single isolate 65 (AY353921) from roots of *P. vulgaris* was considered to be nonpathogenic when it was tested on several legume crops [12,27]. Further investigations are underway in order to determine the possible broader host range of *A. macrosporus*, particularly for crops used in rotations in Sweden.

*A. macrosporus* can cause considerable yield loss in spring barley, which may explain the unusually low yield in certain years with high rainfall after sowing. The large impact on yield has been recorded in field trials in Western Sweden, with 1500 kg/ha in a heavily infested field under wet conditions compared to the expected mean value of 4700 kg/ha (2018–2022) in the area [28]. Crop losses are, however, most prevalent in fields with a high water content, which is a prerequisite for infection of oomycetes in general and especially for *Aphanomyces*.

According to our preliminary results, *A. macrosporus* is widespread in several growing areas in Sweden and Denmark, which indicates that it is not a newly introduced pathogen. The longevity of oospores of *Aphanomyces* also entails a long-lasting problem in crop rotations in soils with a high root rot potential, especially where barley is a common crop. Thus, the discovery of the new pathogenic *Aphanomyces* species in barley, capable of causing significant yield losses, particularly in years with high rainfall, and with a potential host range in economically important crops, may have a great impact on farming systems and crop rotations in Sweden. Further studies are, however, needed to broaden and fulfil our knowledge of this pathogen and of possible means of disease control.

## Figures and Tables

**Table 2:** Mean values, averages from 20 oospores and oogonia per isolate, and ranges of morphometric measurements for *Aphanomyces macrosporus* isolates from barley and spinach, with measurements in micrometres on corn meal agar.

		Oospore Ø			Oogonium Ø		
Isolate	Host	Mean	SEM ^1^	Range	Mean	SEM	Range
*A. macrosporus* B8	Barley	29	0.9	20–36	42	1.8	27–57
*A. macrosporus* B9	Barley	27	1.3	17–40	38	2.1	24–60
*A. macrosporus* B10	Barley	27	0.9	18–35	37	1.6	25–55
*A. macrosporus* B11	Barley	23	0.6	19–31	30	1	23–42
*A. macrosporus* B12	Barley	27	0.8	21–32	37	1.1	29–47
*A. macrosporus* B13	Barley	25	0.5	21–30	34	1	27–47
*A. macrosporus* B14	Barley	26	0.5	22–30	34	0.9	29–45
*A. macrosporus* B15	Barley	28	1.1	18–38	37	1.4	28–54
*A. macrosporus* B17	Barley	24	0.6	19–30	33	1	25–42
*A. macrosporus* B19	Barley	21	0.6	15–24	30	1	23–41
*Aphanomyces* sp. 3 BC ^2^	Barley	20	0.5	15–25	31	0.7	25–42
*Aphanomyces* sp. 3 BD ^2^	Barley	26	1.2	21–30	35	1.5	27–48
*Aphanomyces* sp. 3 C ^2^	Barley	21	1.3	16–24	35	1.5	26–47
*Aphanomyces* sp. 3 D ^2^	Barley	26	0.7	21–31	36	1	26–52
*Aphanomyces* sp. 3 AA ^2^	Barley	22	0.7	18–30	30	0.6	23–42
*Aphanomyces* sp. 3 BA ^2^	Spinach	26	1.2	18–32	35	1.5	27–54
*Aphanomyces* sp. 3 T ^2^	Spinach	25	0.8	21–30	37	1.1	28–54

^1^ SEM: standard error of the mean. ^2^ Data from [11].

## Data Availability

All relevant data are included within the manuscript. Sequence data have been uploaded to GenBank with accession numbers OQ972001–OQ972010, OQ980615, and OQ980616.
